# Identification of novel prognosis-related genes associated with cancer using integrative network analysis

**DOI:** 10.1038/s41598-018-21691-5

**Published:** 2018-02-19

**Authors:** YongKiat Wee, Yining Liu, Jiachun Lu, Xiaoyan Li, Min Zhao

**Affiliations:** 1 School of Science and Engineering, Faculty of Science, Health, Education and Engineering, University of the Sunshine Coast, Queensland, 4558 Australia; 2 The School of Public Health, Institute for Chemical Carcinogenesis, Guangzhou Medical University, 195 Dongfengxi Road, Guangzhou, 510182 China; 3 The School of Public Health, The First Affiliated Hospital, Guangzhou Medical University, Guangzhou, 510120 China; 4 Beijing Anzhen Hospital, Capital Medical University, Beijing Institute of Heart, Lung & Blood Vessel Disease, Beijing, China

## Abstract

Prognosis identifies the seriousness and the chances of survival of a cancer patient. However, it remains a challenge to identify the key cancer genes in prognostic studies. In this study, we collected 2064 genes that were related to prognostic studies by using gene expression measurements curated from published literatures. Among them, 1820 genes were associated with copy number variations (CNVs). The further functional enrichment on 889 genes with frequent copy number gains (CNGs) revealed that these genes were significantly associated with cancer pathways including regulation of cell cycle, cell differentiation and mitogen-activated protein kinase (MAPK) cascade. We further conducted integrative analyses of CNV and their target genes expression using the data from matched tumour samples of The Cancer Genome Atlas (TCGA). Ultimately, 95 key prognosis-related genes were extracted, with concordant CNG events and increased up-regulation in at least 300 tumour samples. These genes, and the number of samples in which they were found, included: *ACTL6A* (399), *ATP6V1C1* (425), *EBAG9* (412), *FADD* (308), *MTDH* (377), and *SENP5* (304). This study provides the first observation of CNV in prognosis-related genes across pan-cancer. The systematic concordance between CNG and up-regulation of gene expression in these novel prognosis-related genes may indicate their prognostic significance.

## Introduction

Prognosis, diagnosis and treatment are key components in medicine. Cancer prognosis involves an assessment of how the disease will affect the individual and an estimation of life expectancy. The objective of prognosis research is to understand and predict the potential outcomes and survival rates^1^. This information would be valuable in clinical trials to identify novel drug agents and improve treatment^2^. Identifying the cancer biomarkers is a crucial part in prognostic studies^3^. Biomarkers are indicators of certain biological conditions^4^ and identifying these has both prognostic and predictive value^5^. A prognostic biomarker provides information concerning the likely outcomes of an individual’s treatment including disease progression and disease recurrence^6^. Examples of prognostic biomarkers are Prostate-Specific Antigen (PSA) in prostate cancer and the phosphatidylinositol 3-kinase (*PIK3CA*) mutation status of tumours - which are associated with human epidermal growth factor receptor 2 (*HER2*) in women with positive metastatic breast cancer^6^. Detection of biomarkers using molecular biology techniques enables the categorisation of molecular signatures of different types of cancer and provides a guide for individual therapy^5^. Biomarkers are also useful for detecting and monitoring the physical changes of a cell during disease progression^4^.

Genetic abnormalities in transcription and translation could serve as prognostic biomarkers in human cancers^7^. Several studies have indicated value of genomic data specifically in relation to gene expression levels as well as clinical prognostic in multifactorial disorders including cancers^8^. These studies also emphasized the importance of personalised medicine and that analysing gene expression signatures may lead to the discovery of novel therapeutic agents for particular cancer types^2^. Currently, gene expression profiling is used to identify gene expression features that correlate with survival following cancer prognosis and this has enabled the creation of expression profiles that can be used to identify the molecular prognosis signature in different types of tumours^9^. Bioinformatics tools have also been developed to identify the molecular signatures^10^ but analysis of data sets across different human cancer types is complex. These data sets can be categorized into several groups including gene expression levels, methylation levels and frequency of genetic mutations^2^ and the results used to build a prognosis model for different group of cancer patients.

Cancer progression involves a series of genetic alterations involving mutations and copy number of variants (CNVs) in human genomes^11,12^. There are two main groups of CNVs: copy number loss (CNL) which is the loss of gene copies; and copy number gain (CNG) which is the addition of gene copies^11^. CNVs are clustered in distinct chromosomal regions and may alter the expression of many different types of genes^13^. In addition, CNVs play a crucial role in the expression for both protein-coding and non-coding genes and can influence and alter the normal signalling pathways^13^. Therefore, it is important to understand the CNVs and their association with gene expression when investigating the disease-associated changes and identifying their significance in cancer prognostic studies.

Several studies have investigated gene expression and CNVs in different cancers^14–16^ but there has been no systematic study of the features of CNVs in prognosis-related genes. We conducted a study to identify the prognosis-related genes using integrative network analysis across different cancer types and their clinical outcomes. We integrated the prognosis-related genes with expression and CNV data, and this will help in identifying the potential biomarkers in multiple cancers.

## Results

### Frequent copy number gain in potential prognosis-related genes across different types of cancer

**Figure 1:**
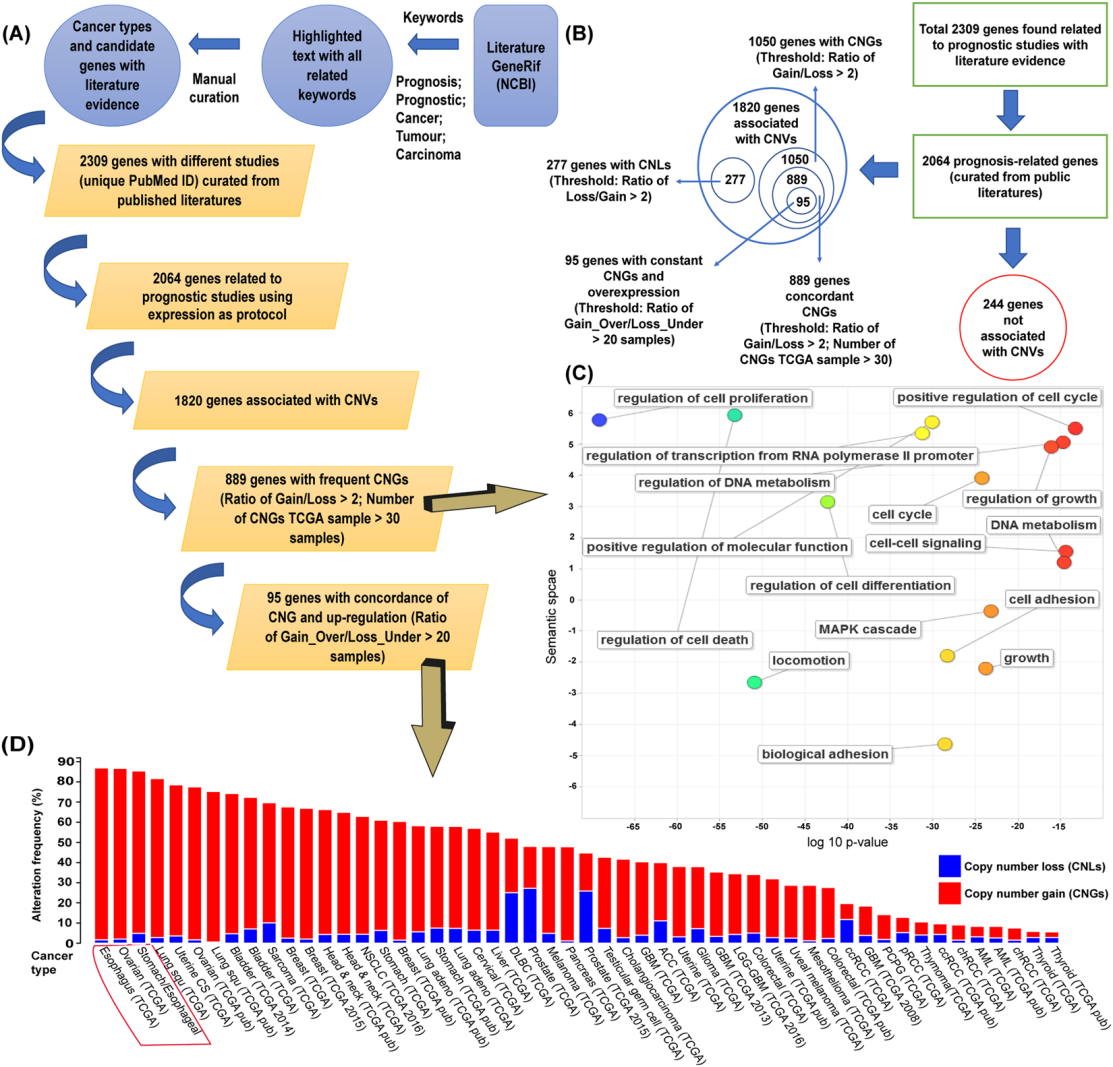
Pipeline for the discovery of consistency in copy number of gain and up-regulation of novel prognosis-related genes in pan-cancer and the gene enrichment analysis of 889 genes with frequent copy number gains (CNGs) and mutational landscape of 95 genes with constant CNGs and up-regulation. (**A**) This flowchart shows the pipeline for finding the novel prognosis-related genes which consistent with the copy number of gain in CNVs and their corresponding gene expression. The work divides into several steps: Identifying short descriptions containing both cancer and prognosis keywords: [(prognosis OR prognostic) AND (cancer OR tumour OR carcinoma)] from GeneRIF (Gene Reference Into Function) database; Manually curating the data from published literature to extract the corresponding gene names in Human. (**B**) 2309 genes with different studies (each with unique PubMed ID) extracted from the literature database and identified 2064 genes related to prognostic studies; A gene set of 1820 genes which associated with CNVs;Total number of 1050 prognosis-related genes identified with frequent CNGs based on the cut-off point (ratio of Gain/Loss > 2)and 277 genes associated with CNLs (ratio of Loss/Gain > 2); 889 genes observed as frequent CNGs with number of CNGs TCGA samples >30; Lastly, 95 genes identified with consistent CNGs and over-expression in the same TCGA samples. (**C**) Gene enrichment analysis of 889 prognosis-related genes with concordant copy number gains (CNGs). The scatterplot presents the summarized GO terms of all 889 prognosis-related genes with CNGs. Circles show the GO clusters and are plotted in two-dimensional space according to other GO terms’ sematic similarities. Y-axis demonstrates the similarity of the GO terms; x-axis indicates the log of corrected P-value (bubbles of right corrected P-values are larger); circle colour represents directly proportional to the frequency of the GO term in the Gene Ontology Annotation (GOA) database (**D**) A general pan-cancer overview between the correlations of copy number variation (CNV) aspects based on 95 prognosis-related genes with up-regulated gene expression conceivably caused by copy number gains (CNGs). Y-axis shows the alteration frequency in percentage (including both amplification and deletion mutation); x-axis indicates the cancer types. Blue - Deletion; Red- Amplification.

To provide an unbiased perspective of CNVs in some major cancer types, our studies were designed based on following the steps as shown indicated in Fig. 1A and the results are given in Fig. 1B. This shows results mapped based on their gene names with concordance CNGs events and up-regulation from the largest cancer genomics data source – TCGA. Most of the genes were identified with CNGs (Fig. 1B) and we focused on those novel prognosis-related genes using expression method only (i.e. each gene with their unique PubMed ID) in prognostic studies and there were 1820 genes associated with CNVs in multiple cancer types (Table S1). We used a defined threshold value of >2 to identify the prevalence of CNVs in these prognosis-related genes by counting the ratio of number of samples with gene copies gain divided by the number samples with gene copies loss and the ratio number of samples with gene copies loss divided by the number samples with gene copies gain. One thousand and fifty prognosis-related genes were identified as CNGs (ratio of Gain/Loss > 2) while 277 genes were associated with CNLs (ratio of Loss/Gain > 2). Finally, 889 prognosis-related genes were observed with frequent CNGs (number of CNGs TCGA samples > 30) and these genes were then used for functional enrichment and integrative analysis (Table S2). We identified that there was a predominance of genes involved in CNGs as 1050 genes were associated with constant CNGs (ratio of Gain/Loss > 2).

Functional enrichment analysis of the 889 genes was conducted using Gene Ontology (GO) terms as functional units (Fig. 1C). The results provide information on enriched with cell cycle, growth, apoptotic process, cell division and cell proliferation: all features related to cancer progression. Cancer results from a single somatic cell that has accumulated multiple DNA mutations and result in cell proliferation caused by mutations in genes that control proliferation and the cell cycle^17^. Abnormal stimulation of the apoptotic process will threaten cell survival and therefore, apoptosis is highly regulated in human cells^18^. Nevertheless, most of the cancerous cells escape this cell death process by disrupting the apoptosis pathway and inactivating pro-apoptotic cell death elements^18^. For example, BCL-2, the first anti-apoptotic gene discovered, is encoded by the human BCL-2 gene and involved in the regulation of programmed cell death including autophagy, necrosis and apoptosis^19^. The elevated level of gene expression of BCL-2 is often found in many cancer types including lung cancer and lymphomas^20^. Overexpression of BCL-2 and related anti-apoptotic proteins has been demonstrated to inhibit cell death induced by growth factor deprivation, hypoxia and oxidative stress^20^. The potential prognosis-related genes with CNG have fundamental roles in chromosome organization^19^. Our enrichment analysis provides insight into the role of these prognosis-related genes in cancer progression including cancer-related pathways, cell growth and cell cycle.

### Correlation of CNG with gene upregulation in novel prognosis-related genes using the corresponding TCGA tumour samples

In order to find novel prognosis-related genes with concordance CNGs and up-regulation, the correlation between CNGs and the overexpression of genes was investigated using the matched TCGA tumour samples. The threshold value (ratio of Gain_Over/Loss_Under) was set at >20 samples and, after investigating the matched TCGA samples for both CNVs gain and gene overexpression, 95 genes were identified with consistent CNGs and gene up-regulation (Table S3). These were identified as potential prognosis-related genes and used for functional enrichment and network analyses. The results from the functional enrichment analysis showed that these genes were related to the cancer progression in the cell cycle (adjusted *P*-value = 1.670E-15) and the biological pathways in cancer (adjusted *P*-value = 1.137E-9). Figure 1D shows the mutational pattern of these genes across different types of cancers and that these genes have a high mutation rate in the tumour samples as shown by gene amplifications. For example, the frequency of genetic alterations in TCGA oesophageal carcinoma that exhibited at least one copy number change for each gene was the highest with 157 cases (85.3%). The frequency of the amplification event in these 95 genes was greater than 84.6% (490 cases) in the ovarian serous cystadenocarcinoma patients. In addition, in TCGA oesophagus-stomach cancers, there were 288 cases (85.4%) with at least one copy number change. More than 80.0% of oesophagus-stomach cancer patients involved gene amplifications. The same proportion of copy number changes in both CNGs and CNLs with more than 60.0% cases were identified in 14 cancer datasets from six types of cancer, including breast cancer, head and neck squamous cell carcinoma, lung cancer, bladder urothelial carcinoma, sarcoma and uterine carcinosarcoma. In addition, there were three cancer types with greater than 70% of CNGs – TCGA lung squamous cell carcinoma (78.8%), uterine carcinosarcoma (75.0%) and ovarian serous cystadenocarcinoma (75.9%). Most of the cancer patients had CNGs, and the deletion events only affected a small group. The results show the significance of features involving the 95 genes that may serve as prognosis-related genes because they promote a large number of copy number gains in clinical applications.

**Figure 2:**
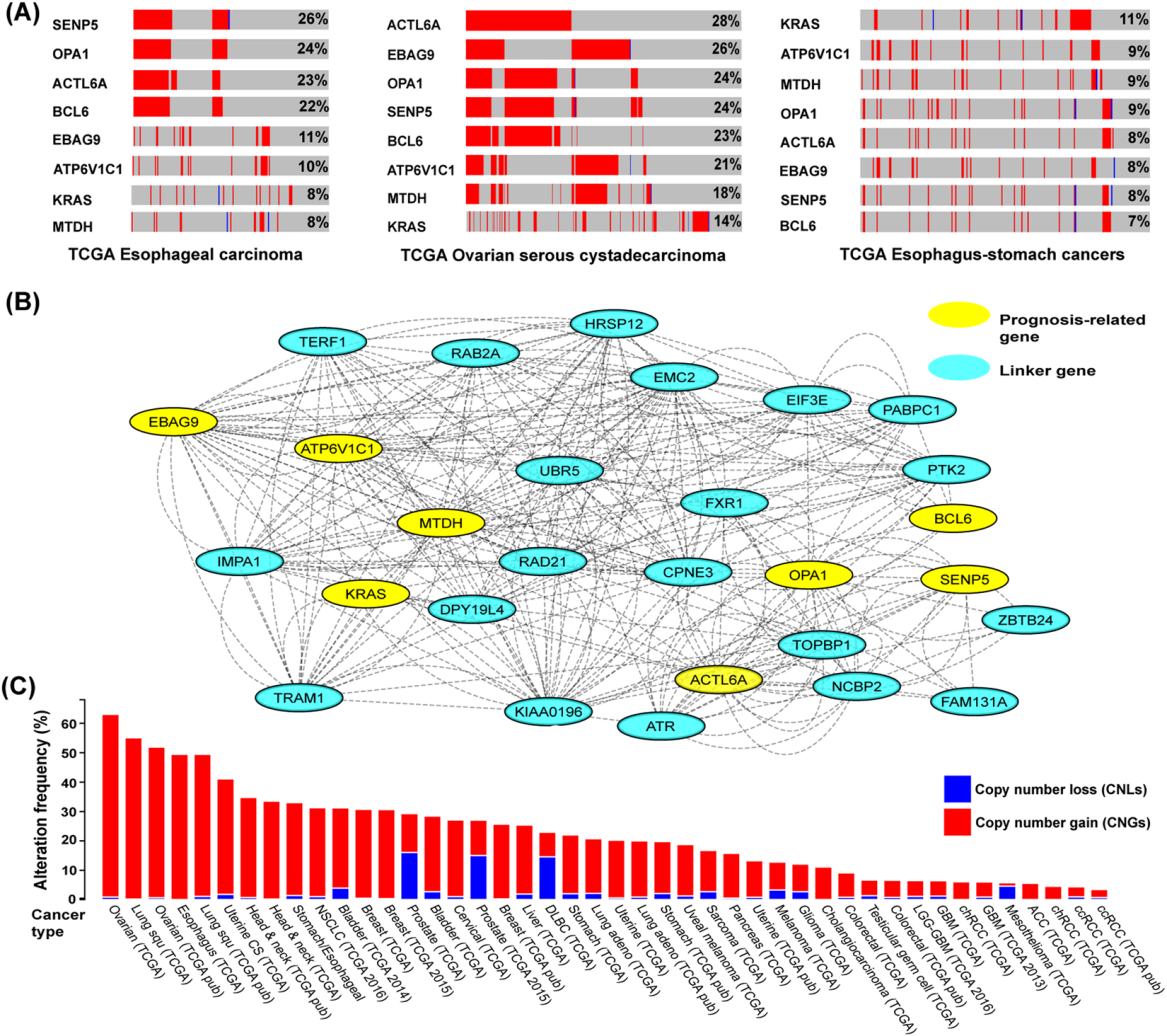
Sample-based mutational and network analysis for the eight-potential cross-cancer prognosis-related genes with high amplification rate. (**A**) Sample-based mutational patterns for the eight genes from the three different cancer samples **-** TCGA esophageal carcinoma, TCGA ovarian serous cystadecarcinoma, TCGA esophagus-stomach cancers. Columns indicate samples and rows indicate genes. The colour bar is used to represent the genomic alterations such as CNVs and somatic mutations. The different mutational types are marked using different colours. The mutational types in (**A**–**D**) were depicted by colours. The red and blue show the amplification and deletion respectively. The grey indicates no mutations in the sample. The percentage represents the alteration frequency for each gene. (**B**) The network of the common eight genes with high amplification rates. The network represents the molecular function-based relationship between these eight genes and the novel linker genes in cancer development. Yellow circles represent prognosis-related genes and blue circles indicate linker genes. (**C**) A pan-cancer global view of copy number variation (CNV) features based on these common eight genes with increased gene expression potentially induced by copy number gains (CNGs). Y-axis shows the alteration frequency in percentage (including both amplification and deletion mutation); x-axis indicates the cancer types. Blue - Deletion; Red- Amplification.

In addition to the sample analysis, we explored the genomic alterations in multiple genes across several tumour samples to further elaborate the prognosis-related genes (Fig. 2). From the sample-based mutational analysis, we selected 20 genes with the highest amplification rates (Table S1) in three different cancer types (with mutation frequency >85.0%): oesophageal carcinoma (87.0%), ovarian serous cystadenocarcinoma (86.7%) and oesophagus-stomach cancers (85.4%). We used the OncoPrint in cBioPortal derived from a query search for alterations in these 95 genes in TCGA oesophageal carcinoma, TCGA ovarian serous cystadenocarcinoma, and TCGA oesophageal-stomach cancers samples. An OncoPrint is a graphical display of gene mutations in human cancer tumour samples. The 20 genes with the highest amplification rate across the three tumour samples were selected (Figure S1, Table S4). From the OncoPrint results in TCGA oesophageal carcinoma, there were six genes with more than 20.0% alteration frequency. Five of them, *FADD*, *SENP5*, *OPA1*, *ACTL6A* and BCL6 showed the highest alteration frequency with >22.0% amplification. In the TCGA ovarian serous cystadenocarcinoma sample, the OncoPrint showed six genes with more than 20.0% of genetic alterations frequency and most of the alterations were related to homozygous addition. *ACTL6A*, *EBAG9*, *OPA1*, *SENP5*, *BCL6* and *ATP6V1C1* had the highest amplifications frequency each with greater than 21.0%. From the OncoPrint of the TCGA oesophageal-stomach cancers sample, a total of seven genes had greater than 10.0% - alterations frequency and those with the highest frequency were: *ERBB2* (25.0%), *JUP* (15.0%), *CUL7* (13.0%), *RAB22A* (12.0%), *CPSF4* (11.0%), *FADD* (11.0%), *IQGAP1* (11.0%), *KRAS* (11.0%) and *LASP1* (10.0%). *SENP5*, *OPA1*, *ACTL6A*, *BCL6*, *EBAG9*, *ATP6V1C1*, *KRAS* and *MTDH* were found in all samples with greater alteration frequency and amplification in comparison with other genes. Sumoylation (SUMO) is a reversible and dynamic post-translational process which is involved in regulating the functions of different proteins including those involved in cellular responses, phosphorylation and protein-protein interactions^21^. Several studies^21^ have reported that the SUMO-specific proteases (SENPs) that remove SUMO from substrates are often amplified in human cancers. For example, *SENP5*, which plays an important role in cell division as well as sustaining the morphology and function of the mitochondria^22^. Findings indicate that breast cancer patients with low expression levels of *SENP5* have a better prognosis than those with high levels^23^. *OPA1* is a member of the dynamin GTPase family and is located in the inner membrane of mitochondria^24^. *OPA1* has a role in regulating cell death, and the cell death signals are amplified due to the formation of an apoptosome when OPA1 interacts with *APAF1* and caspase 9^24^. *OPA1* is overexpressed and has poor prognosis value in lung adenocarcinoma cells^25^. Actin-like 6 A (*ACTL6A*), also known as *BAF53A*, encodes a family member of actin-related proteins (ARPs). *ACTL6A* is commonly involved in activating the transcription process, repressing the selected genes by chromatin remodelling^26^, and plays a key role in lung cancer invasion and metastasis. *ACTL6A* is overexpressed in lung cancer tissues and the upregulation of *ACTL6A* is associated with the clinic-pathological characteristics and is a poor prognostic factor for both cancer types^26^. A protein transcriptional repressor is encoded by *BCL6A* and has been implicated in different types of cancer particularly lymphomas^27^. The role of *BCL6A* in B cell development and lymphomagenesis supports the hypothesis that *BCL6A* plays a major role as a proto-oncogene in lymphoma development^27^. *BCL6A* protein is highly expressed in breast cancer tissues and this expression is correlated with accurate prognosis and poor survival rates for patients^28^. Estrogen receptor-binding fragment-associated antigen 9 (*EBAG9*) is a gene which binds to the estrogen-responsive component located near the 5’-flanking region of the gene. The final product of *EBAG9* is a tumour-associated antigen that is highly expressed in different types of cancer including breast^29^ and kidney^30^. In addition, several studies have indicated that the immunoreactivity of *EBAG9* is positively associated with poor prognosis and its up-regulation is predicted to promote malignant progression in cancers^30^. The function of *Atp6v1c1* in metastasis is poorly defined but studies have shown that Atp6v1c1 is overexpressed in oral cancer patients and encodes an element of vacuolar ATPase (*V-ATPase*), a multi-subunit enzyme that accelerates the process of acidification in the intracellular components of eukaryotic cells^31^. *Atp6v1c1* expression in metastatic oral squamous cell carcinoma indicates that it has a significant role in cancer cell proliferation and metastasis. Our study showed that *Atp6v1c1* may regulate the activity of lysosomal V-ATPase and trigger bone metastasis and breast tumour growth and may be is a promising target in the treatment and control of breast cancer^32^. *KRAS* is a proto-oncogene that encodes a protein member of the small GTPase superfamily. The encoded product binds to the protein which is involved in regulating cellular responses to extracellular stimuli. *KRAS* is the most frequently mutated gene among the RAS gene family and has a 17–25% mutations rate in all cancer types^33^. Most studies show that the *KRAS* gene mutations are poor prognostic factors^33^ but that the upregulation of metadherin (*MTDH*) is associated with tumour progression in female reproductive system cancers. In addition, the overexpression of *MTDH* can predict the survival outcome in female reproductive malignancies^34^. MTDH complies with most of the features that identify the vital elements that regulate numerous processes in carcinogenesis. The expression of MTDH/AEG-1 is up-regulated in different types of cancers including breast and lung cancer. It has been demonstrated that overexpression of MTDH/AEG-1 can trigger the growth of malignant tumours through a complicated oncogenic signalling network^34^. As a result, we identified eight common mutated genes (*EBAG9, MTDH, ATP6V1C1, OPA1, ATCL6A, BCL6, SENP5, KRAS*) in the three different cancer types and used them as cross-cancer biomarkers to perform an integrative network analysis. Most of our results indicated that CNG triggers upregulated expression and is a reliable prognostic marker in cancer prognosis.

### Copy number gain with overexpression in novel prognosis-related genes with the highest number of prognostic studies

**Figure 3:**
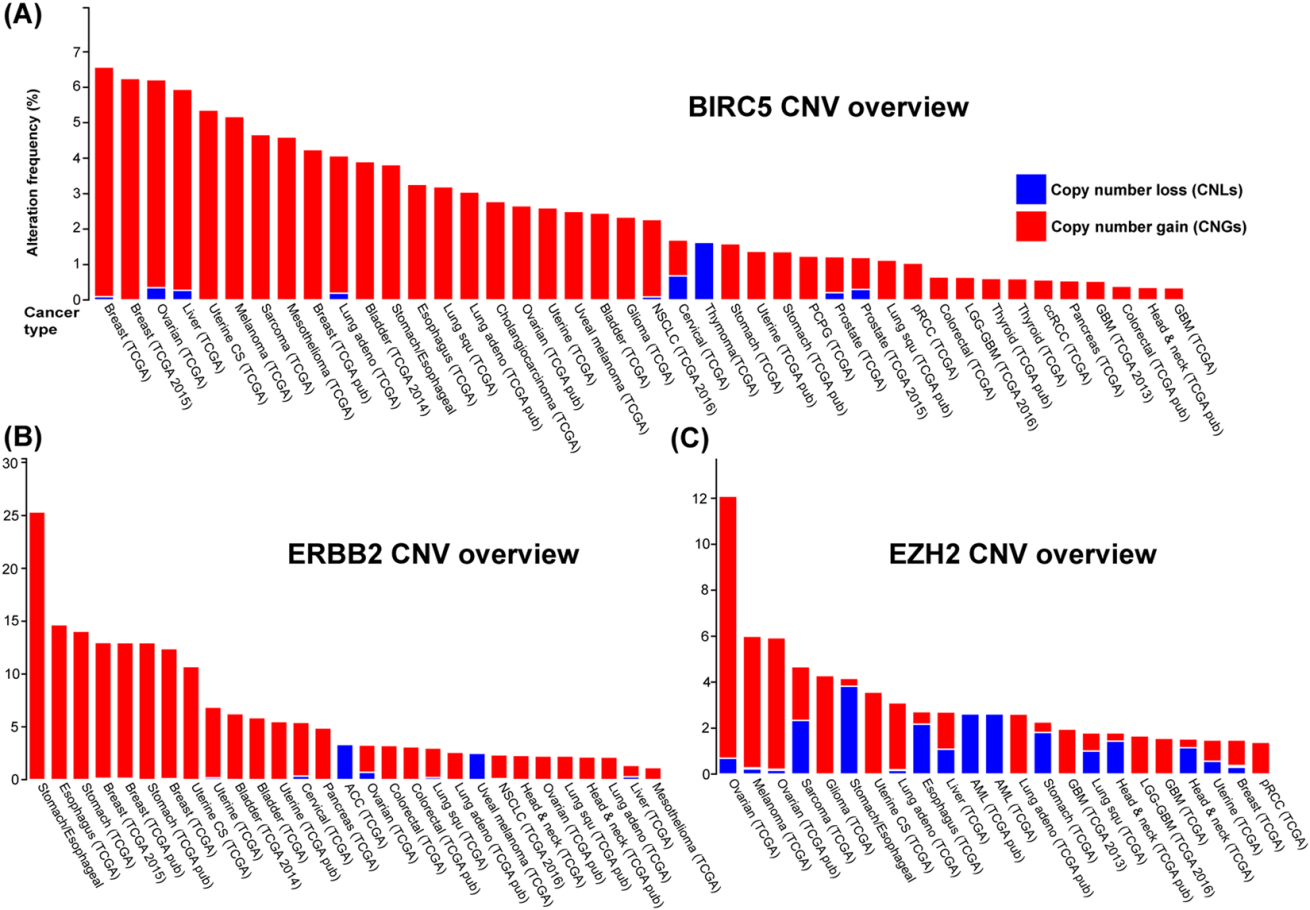
A pan-cancer view of copy number variation (CNV) distribution in three novel prognosis-related genes: *BIRC5* (**A**), *ERBB2* (**B**) and *EZH2* (**C**) and their corresponding CNV mutational landscape. Y-axis shows the mutation frequency in percentage (including both amplification and deletion mutation); x-axis indicates the cancer types. Blue - Deletion; Red- Amplification.

**Figure 4:**
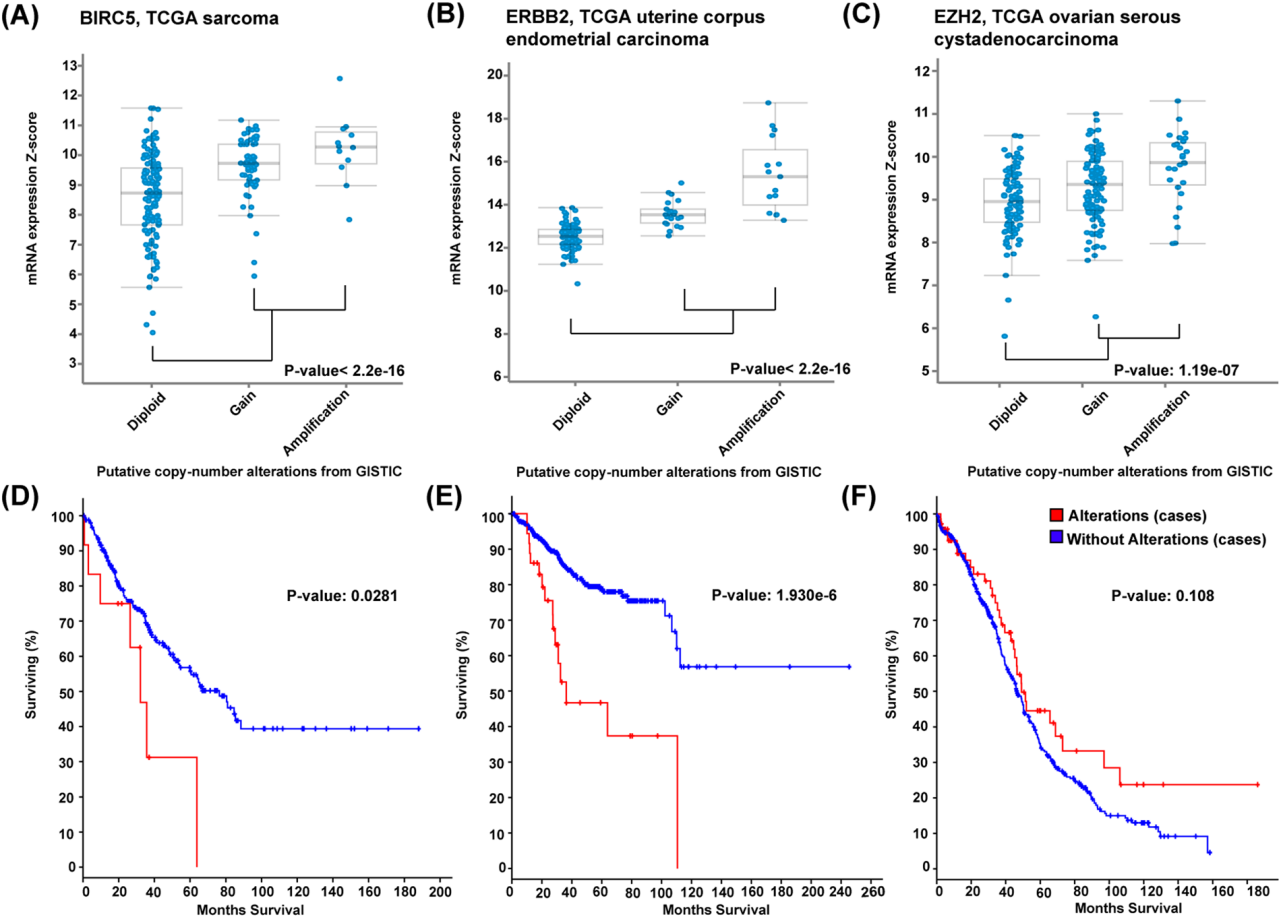
The expression analysis of up-regulated expression of three novel prognosis-related genes with CNGs and their survival curves: *BIRC5*, *ERBB2* and *EZH2*. Plots were derived from cBioPortal based on the Kaplan-Meier analysis. Blue line indicates lower expression and red line indicates higher expression. (**A**) The expression level of BIRC5 in TCGA sarcoma. (**B**) The expression level of ERBB2 in TCGA uterine corpus endometrial carcinoma. (**C**) The expression level of EZH2 in TCGA ovarian serous cystadenocarcinoma. (**D**) Overall survival analysis of BIRC5 in TCGA sarcoma. (**E**) Overall survival analysis of ERBB2 in TCGA uterine corpus endometrial carcinoma. (**F**) Overall survival analysis of EZH2 in TCGA ovarian serous cystadenocarcinoma.

We selected those prognosis-related genes with the highest number of studies (>300) to investigate their CNGs and gene-upregulation. The highest frequency genes in CNGs, *BIRC5*, *ERBB2* and *EZH2* were used to perform a pan-cancer mutational analysis (Fig. 3). *BIRC5* gene is known as a baculoviral inhibitor and inhibits the apoptosis signalling pathway that is expressed in human tissues. This gene has a role in cell cycle regulation including various cell cycle checkpoints^35^. In addition, the expression level of *BIRC5* is found to be associated with tumorigenesis in cancer progression^36^. High copy number of *BIRC5* gene is found in tumour tissues^37^ and several studies have indicated that *BIRC5* is highly amplified in different types of cancer, including pancreatic and lung^35^. *BIRC5* agents have been identified as a potential therapeutic target in cancer treatment but their long-term effectiveness is unclear. This is because there are numerous factors involved in regulating the activity and expression level of BIRC5 which could influence the efficacy of *BIRC5*-targeted therapies^36^. The *ERBB2* oncogene is a member of the epidermal growth factor receptor family that encodes a receptor tyrosine kinase which is usually involved in numerous signal transduction pathways^38^. The overexpression of *ERBB2* has been found in breast tumours and correlates with poor prognosis^38^. In addition, the *ERBB2* gene is overexpressed in lung cancer and prostate cancer. *EZH2* is the key substance of polycomb repressive complex 2 (PRC2) which codes for histone methyltransferase. This enzyme silences the gene through post-translational histone modification^39^ and triggers the oncogenic signalling pathways via chromatin modification and by silencing the tumour suppressor genes^39^. Therefore, histone methyltransferase plays a significant role in oncogenesis. *EZH2* and the production of histone-lysine N-methyltransferase is often highly amplified in various types of human malignancies including lung cancer and breast cancer^40^. Many studies have evaluated whether the overexpression of *EZH2* may be a prognostic factor for survival in patients with lung cancer^40^. The tumour sample with the highest amplification frequency and the most significant overall survival value was selected for each type of cancer. *BIRC5* accounted for 4.6% of amplification frequency of TCGA sarcoma. Another prognosis-related gene, *ERBB2* was amplified in six cases (10.7%) of patients in a uterine corpus endometrial carcinoma dataset. The frequency of gain-of-function in *EZH2* demonstrated a higher percentage in ovarian serous cystadenocarcinoma patients (11.4%, 66 cases) than in skin cutaneous melanoma patients (5.7%, 21 cases). Furthermore, we showed that all these genes were consistently overexpressed in the tumour sample with CNGs (Fig. 4). The frequency of the oncogenes with CNGs and overexpression across the tumour samples suggested that this could be a common mechanism in cancer development. Using cBioPortal, the overall survival rates of patients in these cancer types was compared between tumour samples with or without alterations in each gene, and those which contained the highest number of tumour samples (Fig. 4). Those patients with TCGA uterine corpus endometrial carcinoma had significant overall survival rates with p-value 1.930e-6. We observed that TCGA uterine corpus endometrial carcinoma patients with genetic alterations in *BIRC5* had significantly better overall survival rates when compared to TCGA sarcoma and ovarian serous cystadenocarcinoma patients with gene amplification in *BIRC5* and *EZH2*. The median month survival for sarcoma patients with genetic alterations was 32.13 while that of patients without genetic alterations was 76.35. There was a significantly difference in survival rates between patients with and without genetic alterations. The expressions of *BIRC5*, *ERBB2* and *EZH2* and their mRNA were compared between the cell subsets in the two groups. The expressions data were downloaded from cBioPortal (Fig. 4), statistically analysed using the t test, and compared using the merged data (amplification and gain) and diploid. A P-value of < 0.05 indicated that the difference was statistically significant. We also performed a t-test analysis for each *BIRC5*, *ERBB2* and *EZH2* in TCGA sarcoma, uterine corpus endometrial carcinoma and ovarian serous cystadenocarcinoma. Both *BIRC5* and *ERBB2* genes generated a significant result with P-value < 2.2e-16 in both TCGA sarcoma and uterine corpus endometrial carcinoma. The *EZH2* gene also gave a significant P-value with 1.19e-07 in TCGA ovarian serous cystadenocarcinoma. The difference was statistically significant (P < 0.05) in all the results which suggests that the CNG triggers gene expression changes in these potential prognosis-related genes. Our results indicate that these changes could be an important factor in examining and predicting the outcome of a disease including cancers.

To identify the expression of the eight-potential cross-cancer biomarkers in the prognosis of four different cancer types we used to show in the Kaplan-Meier Plotter online platform (www.kmplot.com) namely breast^41^, ovarian^42^, lung^43^ and gastric^44^ cancer. We evaluated all the eight prognostic-related genes to examine their impact in the recurrence-free survival (RFS) of the four different cancer type patients. The desired Affymetrix was valid: 202666_s_at (ACTL6A), 226463_at (ATP6V1C1), 203140_at (BCL6), 204274_at (EBAG9), 204010_s_at (KRAS), 212248_at (MTDH), 216071_x_at (OPA1) and 213184_at (SENP5). Survival curves were plotted for all patients in breast (n = 1015; Figure S2), ovarian (n = 1816; Figure S3), lung (n = 2457; Figure S4) and gastric (n = 1815; Figure S5) cancer. When group of patients was divided into four groups according to the different cancer types, half of the genes: (i) ACTL6A (P = 2.3e-15 in breast cancer, P = 8e-04 in ovarian cancer, P = 0.00016 in lung cancer and P = 2.3e-15 in gastric cancer), (ii) ATP6V1C1 (P = 0.014 in breast cancer, P = 0.024 in ovarian cancer, P = 1.3e-06 in lung cancer and P = 0.014 in gastric cancer), (iii) BCL6 (P = 0.031 in breast cancer, P = 0.00058 in ovarian cancer, P = 0.028 in lung cancer and P = 0.031 in gastric cancer) and (iv) EBAG9 (P = 1.4e-09 in breast cancer, P = 0.08 in ovarian cancer, P = 0.00029 in lung cancer and P = 1.4e-09 in gastric cancer) were associated with RFS. Interestingly, we identified that MTDH was not statistically associated with RFS in ovarian cancer (P = 0.59); however, the high expression of MTDH was associated with a poor prognosis in other three cancer types – breast (P = 3.5e-10), lung (P = 2.1e-06) and gastric (P = 3.5e-10; Sup Fig. 1). The high expression of OPA1 showed statistically significant with P < 0.05 in both breast and gastric cancer (P = 1.6e-06, respectively); while SENP5 was associated with RFS in the other two cancer types – ovarian cancer (P = 0.011) and gastric cancer (P = 1.1e-05). Overall, these results will help to further validate the reliability and reproducibility of these eight prognostic genes and may aid in assessing the patients’ risk profile.

### Network connectivity of potential prognostic marker and oncogene with high frequency of gene amplification and overexpression

We performed network analysis using GeneMANIA and Cytoscape to identify the correlation among the eight genes identified from our expression analyses of genes with both high frequency CNGs and consistent gene up-regulation. The derived network (Fig. 2B) comprised of eight core genes and another 20 that were shown in Cystoscape. Genes (nodes) with the highest number of interactions were *EBAG9* (17 connections), *MTDH* (16), *ATP6V1C1* (14), *OPA1* (11), *ATCL6A* (7), *BCL6* (6), *SENP5* (5) and *KRAS* (3). These 28 genes have been implicated in several biological and cellular processes including cell-cell junction and cell-cell junction assembly. Using Toppfun, the functional enrichment results show that these 20 genes are enriched with regulation of translation and cell aging. Translational regulation has been shown to play an important role in cancer and tumour progression. Tumour cells use these alternative mechanisms of translation initiation to promote survival during tumour progression^45^. Cellular senescence is a mechanism of cellular aging that has diverse effects on both cancer and tissue aging. After a certain cell division, primary human cells permanently lose their ability to proliferate, resulting in a senescent phenotype in which major changes take place in various cellular phenotypes and epigenomes^46^. Because senescent cells are defined by their inability to proliferate and constitute a barrier against tumour formation, an epidemiologic link between aging and cancer was hypothesized^46^. The genes involved in cell aging are *TERF1*, *OPA1* and *ATR*. We performed integrative analysis of the linker genes (*KIAA0196*, *HRSP12*, *CPNE3*, *IMPA1*, *FAM131A*, *NCBP2*, *DPY19L4*, *FXR1*, *RAD21*, *EMC2*, *TERF1*, *TOPBP1*, *UBR5*, *EIF3E*, *TRAM1*, *PTK2*, *ATR*, *RAB2A*, *PABPC1* and *ZBTB24*) which were identified in GeneMANIA using cBioPortal. The results show a significant amplification frequency in all the tumour samples (Fig. 2C). The cases with more than 40.0% genetic alterations and including both CNLs and CNGs were identified in six cancer datasets from four cancer types: ovarian serous cystadenocarcinoma, lung squamous cell carcinoma, oesophageal carcinoma and uterine carcinosarcoma. For example, the TCGA ovarian serous cystadenocarcinoma patients had more than 60.0% (360 cases) genetic alteration in CNGs. In particular, TCGA ovarian serous cystadenocarcinoma patients had significant overall survival rates of a p-value 0.0351. The median month survival for ovarian serous cystadenocarcinoma patients with genetic alterations was 48.72, while that of patients without genetic mutation was 39.55. Overall, most of cancer cohort patients had CNGs compared to patients affected with CNLs. This implied that these linker genes also play a significance role in prognostic studies through genetic alterations in high frequency of copy number gains.

## Conclusion

This study has revealed some significant somatic mutational characteristics of prognosis-related genes in multiple cancer types, particularly with respect to the CNVs and their effects on gene expression. The results revealed that most of the prognosis genes were associated with CNGs and, therefore, we focused on the concordant patterns between CNG and gene up-regulation. Our results provided information on the correlation between gene dosage and somatic CNV in prognosis genes but a more systematic examination of the expression quantitative trait locus would provide detailed information on the relationship between CNV and gene expression. In addition, this study showed that these prognosis-related genes were associated with cancer pathways including the MAPK cascade. From the OncoPrint analysis of 95 oncogenes, we observed that there are eight oncogenes with high amplification rate in TCGA ovarian serous cystadenocarcinoma, TCGA oesophageal carcinoma and TCGA lung squamous cell carcinoma. The results indicate that these eight oncogenes – *EBAG9*, *MTDH*, *ATP6V1C1*, *OPA1*, *ATCL6A*, *BCL6*, *SENP5* and *KRAS* are likely to be important cross- cancer target genes for cancer therapies and may also be associated with the patient’s survival rate. Further experimental analysis and validation may provide insight into the potential molecular mechanisms underlying copy number gain and recurrent over-expression. However, the limited sample size in some of the cancer types may remove many CNVs with lower frequencies. In addition, the signals outside the pre-designed probes may be lost as TCGA largely depends on the CGH array between normal and cancer samples for distinguishing different types of CNVs. This causes in limited sample sizes and indicates the presence of many undiscovered structural variants in cancer development.

Our systematic investigation of copy number variations in potential prognosis-related genes showed that the copy number gain of the prognosis-related genes clustered in several regions. These genes obtained from prognostic studies using expression experimental method were associated with copy number gain and have significant roles in cancer-related pathways. The gain of copy number in these prognosis-related genes may promote the gene expression change associated with tumorigenesis. Given the large amount of information that CNVs can provide with regard to clinicopathological characteristics and complex disease signalling patterns, their use in future explorations in prognostic studies will facilitate the discovery of novel biomarker and drug agents to improve patient preselection for clinical trials.

## Methods

### Cancer prognosis-related gene expression changes curated from published literature

To examine cancer prognosis-related genes globally, we conducted an extensive literature search and curation. By using Perl regular expression, we identified short descriptions containing both cancer and prognosis keywords: [(prognosis OR prognostic) AND (cancer OR tumour OR carcinoma)] from GeneRIF (Gene Reference Into Function) database (October, 2016). The data were manually curated from published literature to extract the corresponding gene names in Human. There were 2370 genes with different studies (each with unique PubMed ID) extracted from the literature database and we identified 2064 genes related to prognostic studies. We focused on those prognostic studies which related to gene expression measurements. To systematically investigate the somatic CNVs in novel prognosis-related genes, we developed a pipeline and generated a list of 1820 genes which are associated with CNVs (Table S1).

### Pan-cancer CNV data for prognosis-related genes from The Cancer Genome Atlas (TCGA)

To explore the global view of CNVs in several major types of cancer in an unbiased way, we overlapped all these 2064 prognosis-related genes with the somatic CNVs determined from TCGA CNV data from the Catalogue of Somatic Mutations in Cancer (COSMIC) database (V73)^47^, which is one of the largest resources for cancer genomics research. It resulted 1820 genes were associated with CNVs. The number of TCGA samples with gain or loss copies were counted, and we defined a threshold value to prioritize the instructive CNV occurrences for these prognosis-related genes. Particularly, we set two cut-off values with ratio of Gain/Loss (at least twice of TCGA samples with CNGs as TCGA samples with CNLs) and ratio of Loss/Gain (at least twice of TCGA samples with CNLs as TCGA samples with CNGs) >2 to determine the prevalence of CNVs in these prognosis-related genes. This approach resulted in 1050 prognosis-related genes with the evidence of an overall gain of CNVs and 277 prognosis-related genes were associated with CNLs. Since there were more than half of the prognosis-related genes were found to be CNGs, we selected those prognosis-related genes with higher frequency of CNGs. We focused on those 1050 prognosis-related genes with more than 30 TCGA samples with CNGs and we further identified 889 genes with frequent CNGs. These genes were used to perform gene expression analysis.

### Gene expression analysis of prognosis-related genes with frequent CNGs

To examine the correlation between the CNVs and gene expression changes of the 889 prognosis-related genes with frequent CNGs, we incorporated their gene expression changes in the matched TCGA samples using gene expression data. Among these, we identified that there was a predominance of genes involved in CNGs, therefore we only focused on those gene expression changes in the matched TCGA samples with CNGs and over-expression. We counted the number of identical TCGA samples in both CNVs and expression data (CNGs and over-expression) for each gene. The Z-score of the expression data was applied to identify whether these genes are over-expressed or under-expressed in a sample. In detail, a Z-score refers to the standard deviations away from the mean of expression in the reference, and the equation (1) is shown as below where x represents the expression in tumour sample; µ represents the mean expression in all the samples and σ represents the standard deviation of expression in reference samples:

1 $$Z=\frac{x-\mu }{\sigma }$$

We used the Z-score threshold value 2 to determine the up-regulated prognosis-related genes in specific TCGA samples. In this research, we focused on the prognostic studies that used expression as the protocol of the experiments. The number of samples with consistent over-expression and CNGs were calculated for each gene. The threshold value (ratio of Gain_Over/Loss_Under) was set to >20 samples and 95 genes were generated with consistent CNGs and over-expression. The main reason for this was to identify a reliable gene list with constant CNG and over-expression. We set different cut-off values and we managed to narrow down the gene list to less than 100 genes. Therefore, this level of gene list would be performed better for functional analysis. To examine the CNVs patterns in TCGA samples, the integrative analysis was performed using a free web database known as cBioPortal (http://cbioportal.org)^48^. The cBioPortal for Cancer Genomics allow users to explore, analyse and visualize the multidimensional cancer genomics data. In addition, the web portal provided information for the tumour samples from The Cancer Genome Atlas (TCGA) and the International Cancer Genome Consortium (ICGC). The list of 95 genes was used to explore their corresponding CNVs expression plots in the TCGA samples using cBioPortal.

### Functional enrichment and network analysis

To investigate the related biological systems in the prognosis-related genes with concordance CNGs events and gene over-expression, we analysed the results using the online tools ToppFun^49^, REVIGO^50^ and GeneMania^51^. The molecular functions of the 95 prognosis-related genes were analysed using Toppfun. Toppfun is a web database which allows users to explore the molecular functions of gene ontology (GO), cellular components, biological processes and pathways. From the Toppfun results, a total of 50 enriched GO terms was generated and we extracted their IDs and corresponding p-values for the visualization process using REVIGO (http://revigo.irb.hr/). REVIGO summarized and removed the redundant GO terms from a long list. The GO results served as input data in REVIGO and it produced a semantic similarity-based scatterplot of GO terms from Toppfun. To perform the network analysis, we used GeneMania to identify the interactions of the selected genes. We then utilised Cytoscape to characterize and visualise the network results generated from GeneMania.

## Electronic supplementary material

Suppplementary information

Table S1

Table S2

Table S3

Table S4

Table S5
