# Supplementary material for: Identification of novel prognosis-related genes associated with cancer using integrative network analysis
Source: Sci Rep. 2018 Feb 19;8:3233. doi: 10.1038/s41598-018-21691-5 (PMC5818516; doi:10.1038/s41598-018-21691-5)
Supplement: Supplementary file 1 — Suppplementary information [file 41598_2018_21691_MOESM1_ESM.pdf]

SUBJECT AREAS

Systems biology
Cancer genomics
Prognosis
Oncogenes
Pathway analysis

Identification of novel prognosis-related genes associated with cancer using integrative network analysis

YongKiat Wee[1], Yining Liu[2], Jiachun Lu[2,3], Xiaoyan Li[4§], Min Zhao[1§]

[1]School of Science and Engineering, Faculty of Science, Health, Education and Engineering, University of the Sunshine Coast, Queensland, 4558, Australia.

[2]The School of Public Health, Institute for Chemical Carcinogenesis, Guangzhou Medical University, 195 Dongfengxi Road, Guangzhou 510182, China.

[3]The School of Public Health, The First Affiliated Hospital, Guangzhou Medical University, Guangzhou 510120, China.

[4]Beijing Anzhen Hospital, Capital Medical University; Beijing Institute of Heart, Lung & Blood Vessel Disease; Beijing, China.

§ To whom correspondence should be addressed, xiaoyanli82@163.com, mzhao@usc.edu.au

Supplement

Table S1. 1820 prognosis-related genes associated with CNVs.

Table S2. 889 prognosis-related genes with frequent CNGs.

Table S3. 95 prognosis-related genes with concordant CNGs and over-expression.

Table S4. 20 genes with high amplification rates.

Table S5. KM plots of eight potential cross-cancer genes in breast, lung, ovarian and gastric cancer.

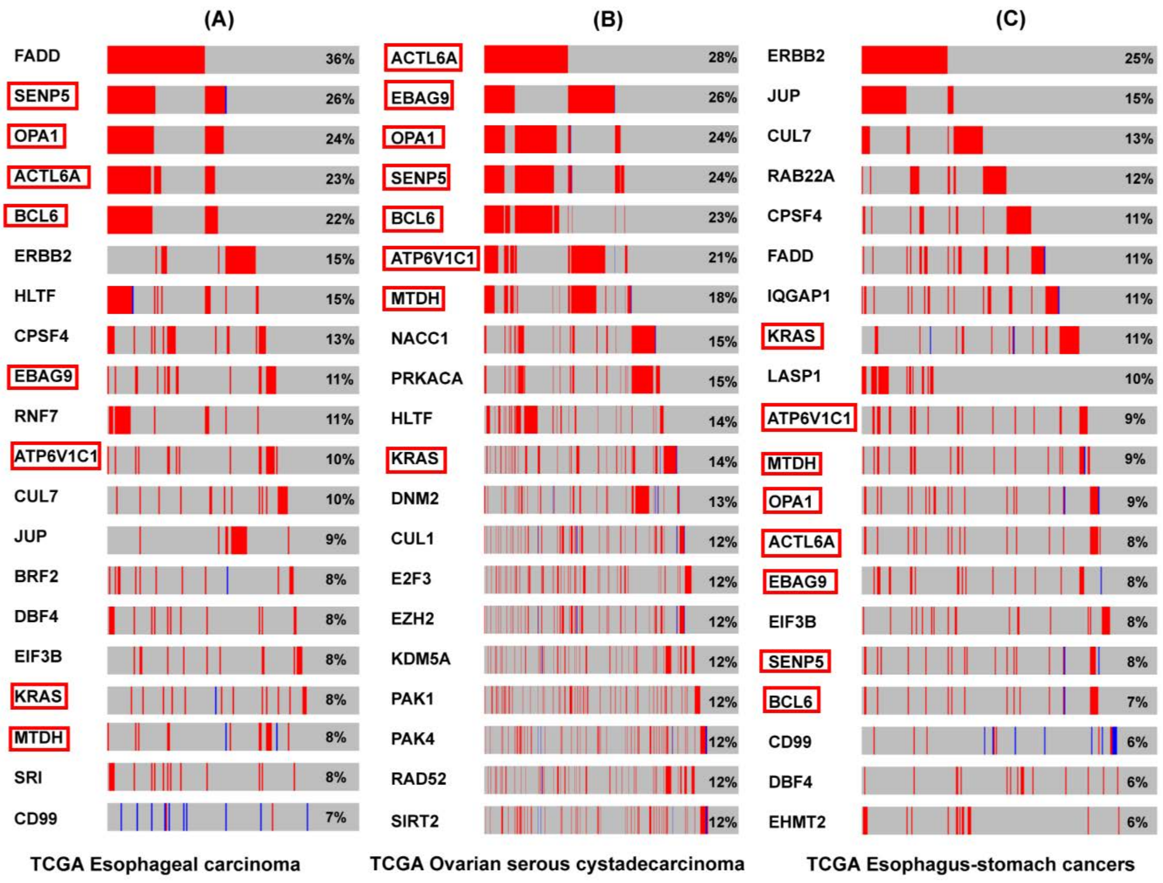

Figure S1. Sample-based mutational analysis of 20 genes with high amplification rate in three different cancer types. (A) Esophageal carcinoma (B) Ovarian serous cystadenocarcinoma (C) Esophagus-stomach cancers.

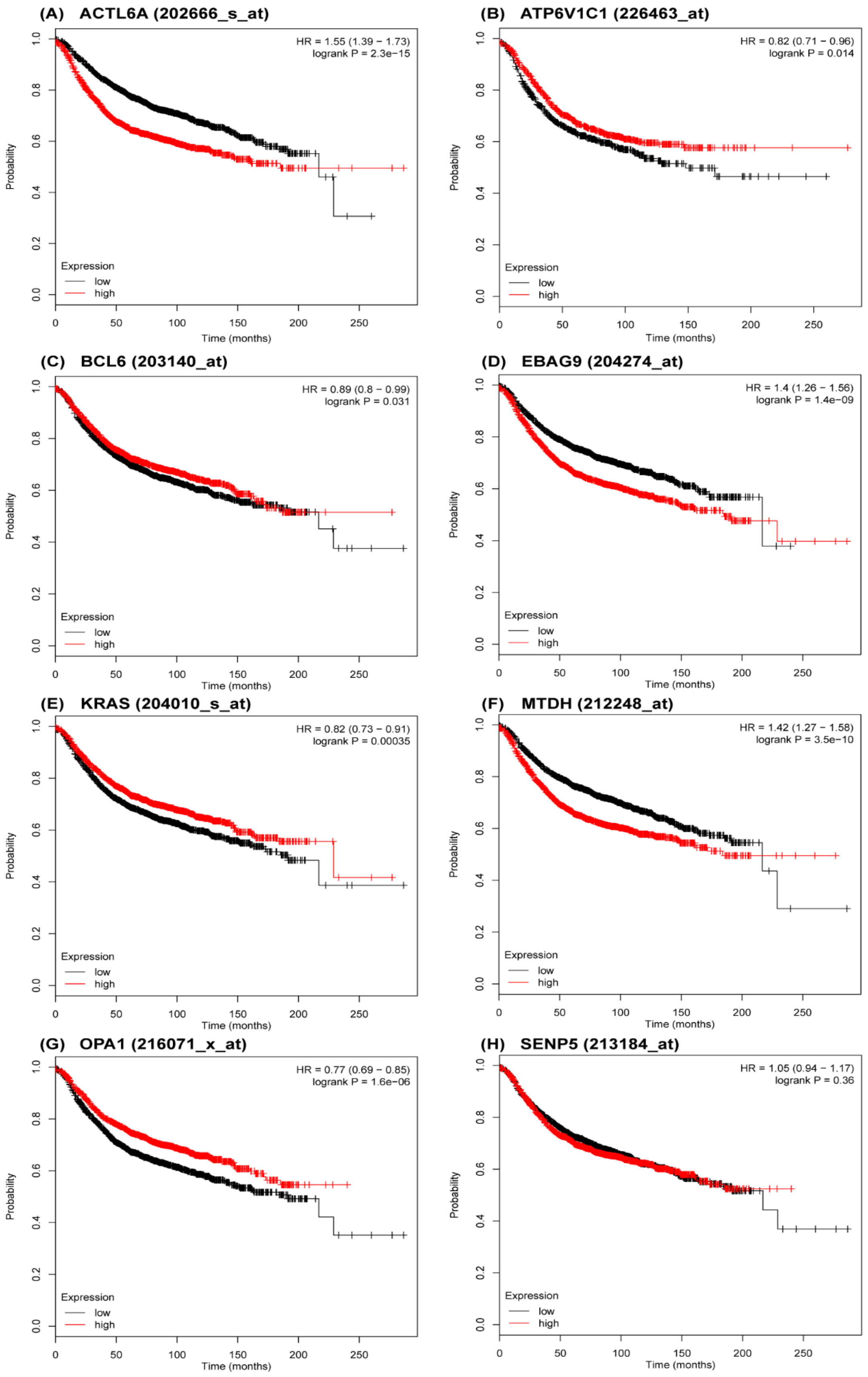

Figure S2. The prognostic value of the eight-potential cross-cancer gene expression in breast cancer patients. (A) ACTL6A (202666_s_at) (B) ATP6V1C1 (226463_at) (C) BCL6 (203140_at) (D) EBAG9 (204274_at) (E) KRAS (204010_s_at) (F) MTDH (212248_at) (G) OPA1 (216071_x_at) (H) SENP5 (213184_at).

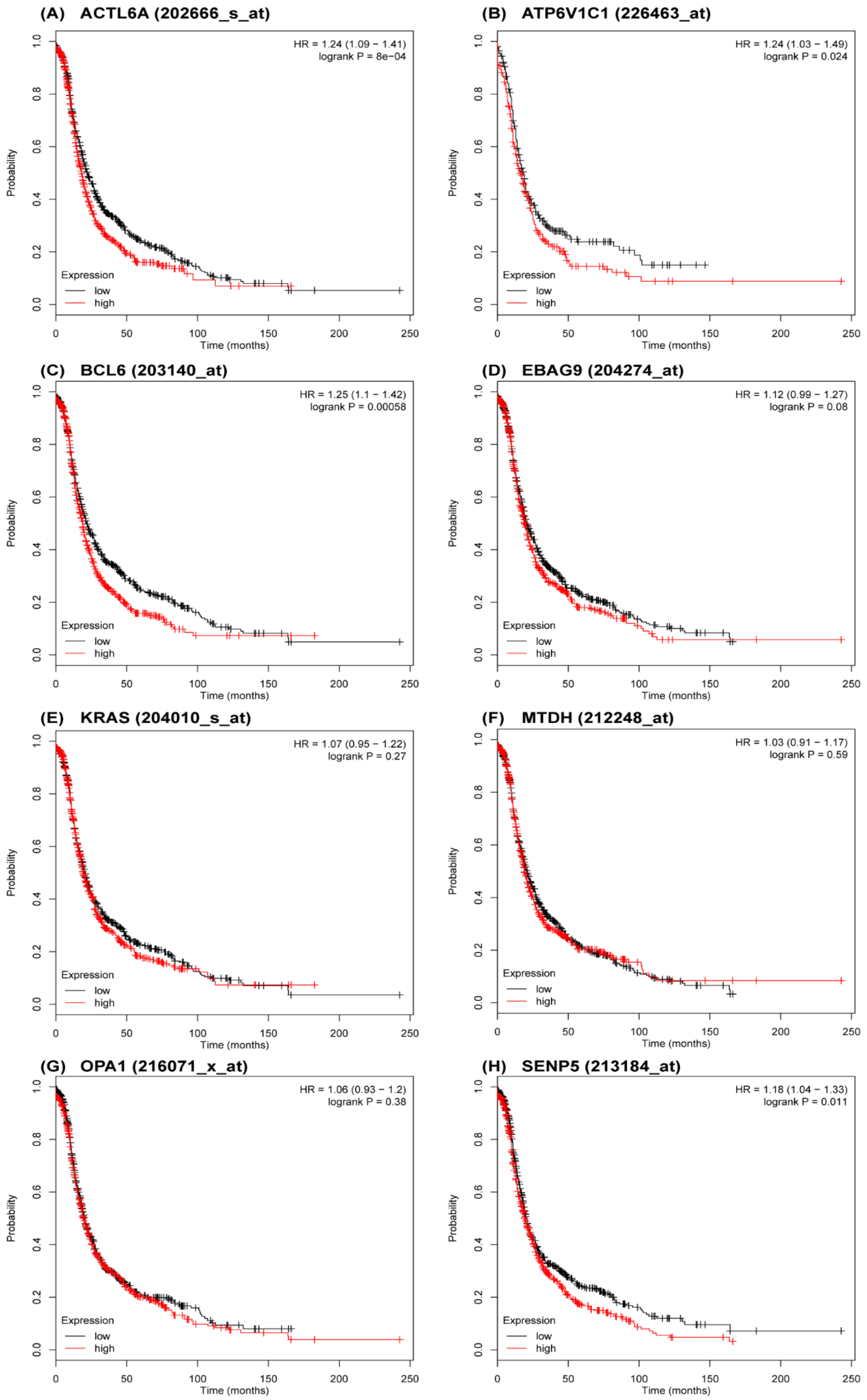

Figure S3. The prognostic value of the eight-potential cross-cancer gene expression in ovarian cancer patients. (A) ACTL6A (202666_s_at) (B) ATP6V1C1 (226463_at) (C) BCL6 (203140_at) (D) EBAG9 (204274_at) (E) KRAS (204010_s_at) (F) MTDH (212248_at) (G) OPA1 (216071_x_at) (H) SENP5 (213184_at).

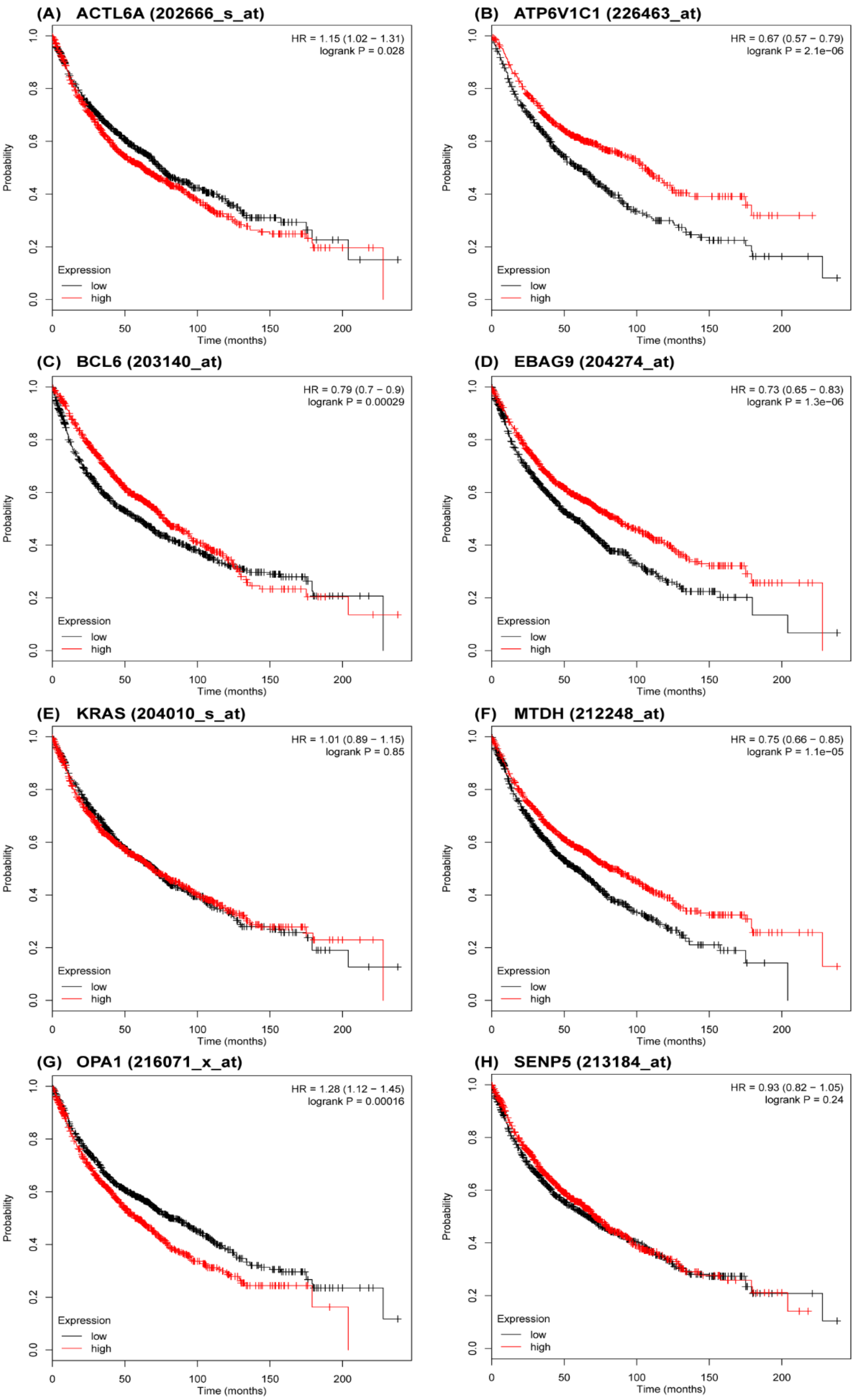

Figure S4. The prognostic value of the eight-potential cross-cancer gene expression in lung cancer patients. (A) ACTL6A (202666_s_at) (B) ATP6V1C1 (226463_at) (C) BCL6 (203140_at) (D) EBAG9 (204274_at) (E) KRAS (204010_s_at) (F) MTDH (212248_at) (G) OPA1 (216071_x_at) (H) SENP5 (213184_at).

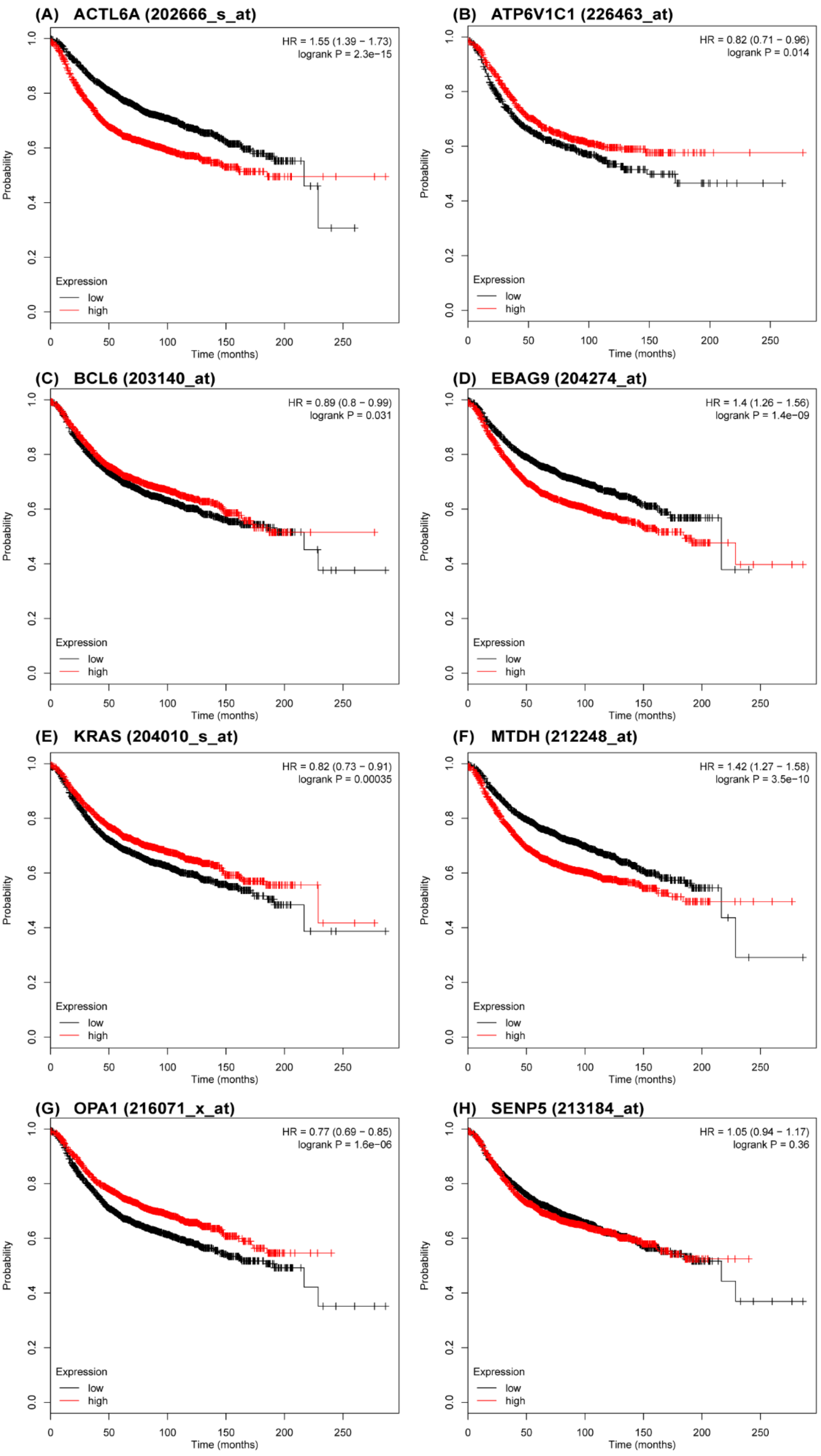

Figure S5. The prognostic value of the eight-potential cross-cancer gene expression in gastric cancer patients. (A) ACTL6A (202666_s_at) (B) ATP6V1C1 (226463_at) (C) BCL6 (203140_at) (D) EBAG9 (204274_at) (E) KRAS (204010_s_at) (F) MTDH (212248_at) (G) OPA1 (216071_x_at) (H) SENP5 (213184_at).
